# Ventricular tachycardia because of myocardial infarction after COVID‐19 vaccination

**DOI:** 10.1002/joa3.12771

**Published:** 2022-08-19

**Authors:** Dan‐Ying Lee, Chin‐Yu Lin, Shao‐Sung Huang

**Affiliations:** ^1^ National Yang Ming Chiao Tung University Hsinchu Taiwan; ^2^ Department of Cardiovascular Disease Taipei Veterans General Hospital Taipei Taiwan

**Keywords:** COVID‐19, myocardial infarction, vaccine, ventricular tachycardia

This case report presents a myocardial infarction (MI) after the AstraZeneca (AZ) coronavirus disease 2019 (COVID‐19) vaccination and raises a safety concern about the patients with underlying coronary artery disease (CAD) to get the vaccination.

We report a case of an 85‐year‐old man with a medical history of end‐stage renal disease with regular hemodialysis. He received catheter ablation for sustained right ventricular outflow tract ventricular tachycardia (VT) 6 months before this admission. The perioperative coronary artery angiography (CAG) demonstrated insignificant CAD (Figure 1A). He visited the clinic of cardiology with chest discomfort, palpitation, and dyspnea after 1 week of AZ COVID‐19 vaccination. He had no other symptoms, specifically no fever or symptoms of upper respiratory infection. He was also a non‐smoker. The follow‐up 24‐h Holter detected several episodes of non‐sustained and sustained VT (max rate: 151 bpm). The QRS morphology of the VT is different from prior VT originating from the right ventricular outflow tract. As a result of the concern of vascular side effects of the vaccine, repeated CAG was arranged. On examination, the COVID‐19 polymerase chain reaction testing was negative. The lab data showed normal electrolytes, thyroid function, and digoxin level. However, the troponin‐I level showed mild elevation (0.16 ng/ml), the D‐dimer level is higher than 4 times the upper limit of normal (2.438 μg/ml), and his platelet level decreased from 278 to 110 10^3^/μl. The 12‐lead electrocardiogram (ECG) showed sinus rhythm with the initiation of ventricular tachycardia and new subtle q waves in lead I and aVL (Figure 2). The VPC morphology was nonuniform. The dominant VT morphology has a right axis and right bundle branch block pattern which originated from the left ventricular. The follow‐up transthoracic echocardiogram showed decreased left ventricular ejection fraction from 59% to 37% with diffuse hypokinesis. The CAG revealed total occlusion of the left circumflex artery (LCX) without collateral circulation from the left anterior descending artery (Figure 1B) and right coronary artery (Figure 1C), which confirmed the diagnosis of a recent MI. During admission, enzyme‐linked immunosorbent assay (ELISA) of Anti‐Human Heparin Platelet Factor 4 (HPF‐4) antibody was checked but the result is negative (Optical Density value: 0.229, reference: positive if OD > 0.4). The possibility of vaccine‐induced thrombosis and thrombocytopenia (VITT)‐related myocardial infarction still could not be excluded. During the percutaneous intervention, thrombosis aspiration was performed with an Export AP 6 Fr aspiration catheter, and thrombosis in the LCX occlusion was identified and removed. After a successful percutaneous intervention, the symptom was relieved. However, an episode of sustained VT with unstable hemodynamics for 3 days was documented after revascularization and terminated spontaneously 5 days later. ICD was implanted for secondary prevention after a discussion with the family. The spontaneous recovery of platelet level was noted before discharge.

**FIGURE 1:**
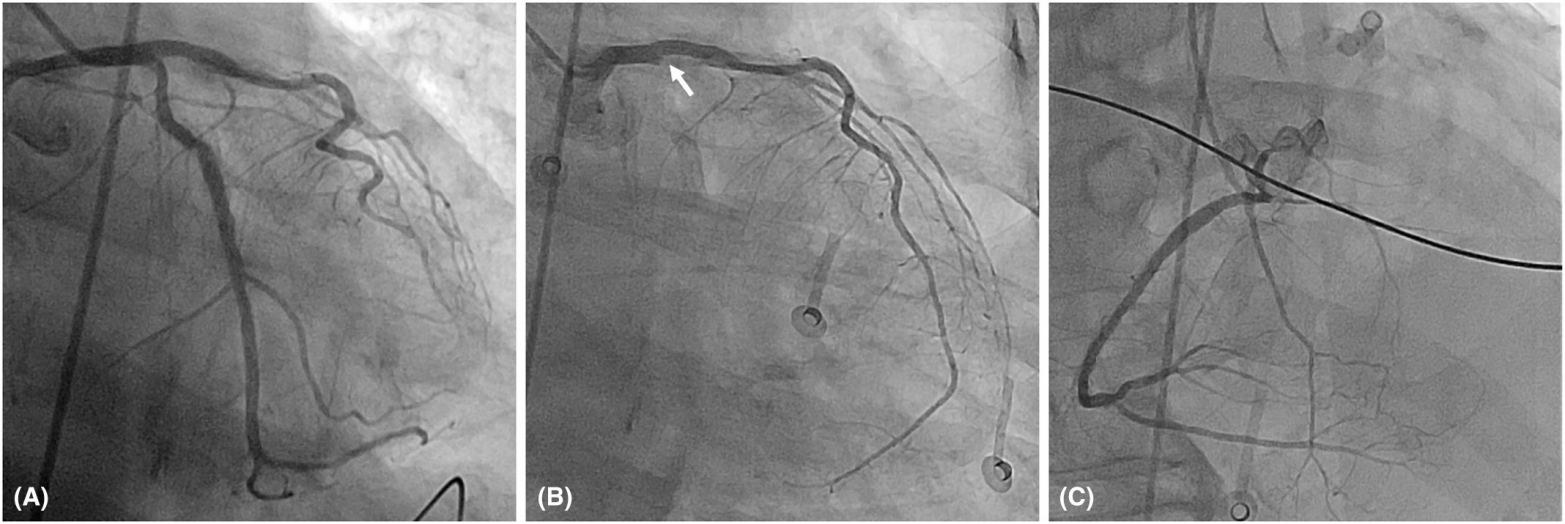
Coronary artery angiography of the patient demonstrates: (A) An insignificant coronary artery disease with patent left circumflex artery 6 months ago. (B) A total occlusion at the ostium of the left circumflex artery without collateral from the left anterior descending artery (white arrow) after vaccination. (C) No collateral from the right coronary artery.

**FIGURE 2:**
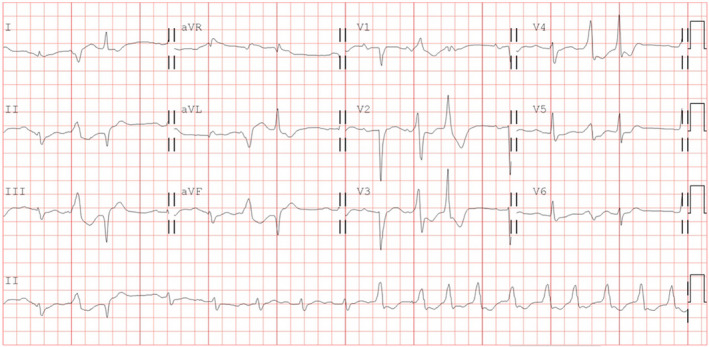
Electrocardiogram of the patient represents sinus rhythm with polymorphic ventricular tachycardia.

Based on the ACC statement, the studies from the United States demonstrate atherosclerosis cardiovascular disease (ASCVD) was associated with adverse outcomes with COVID‐19 infection. Given this collective evidence, older patients with multiple comorbidities, including CV conditions, and/or frailty should be considered at high risk and thus prioritized for COVID‐19 vaccination.

To date, three COVID‐19 vaccines, Pfizer/BioNTech, AstraZeneca, and Moderna, are currently being used in Taiwan. All vaccines and medicines have some side effects. The most well‐published and correlated adverse reaction nationwide is a condition known as vaccine‐induced thrombosis and thrombocytopenia (VITT).^1^ Taiwan has also recorded the first case of VITT after 500 577 doses of the AstraZeneca vaccine that have been administered. Although most cases of VITT resulted in venous clots, arterial clots have also been reported which could lead to chest pain if it affects the coronary artery.

The AZ Vaccine is based on a replication‐incompetent chimpanzee adenovirus vector that expresses the spike protein, which was evaluated in clinical trials involving more than 23 000 participants. The most frequently reported adverse reactions in these trials were injection‐site tenderness, headache, fatigue, myalgia, malaise, pyrexia, chills, arthralgia, and nausea. So far, only VITT was confirmed to be connected to AZ vaccination. There are still a few potential serious adverse side effects such as MI after vaccination reported. Chatterjee, S. et al. reported a case of a 63‐year‐old man who developed MI after AZ vaccination,^2^ and Boivin et al. report another case of a 96‐year‐old female with poorly controlled hypertension, who suffered a MI just 1 h after her first Moderna vaccination.^3^ Both of them did not receive cardiac catheterization due to a lack of facility for PCI and refusion by patients, respectively. We present the first case with the image proof of myocardial infarction after the vaccination. After comparing the CAGs before and after vaccination with an interval of 6 months, our case illustrates the possibility of vaccination to trigger cardiac complications in patients with underlying coronary artery disease. The mechanism of MI after vaccination is still uncertain. It can be ischemia caused by the mismatch of supply and demand or vasospastic allergic response by the coronary artery.^4^ Based on the clinical and post‐MI analysis, VITT could not be confirmed by the negative anti‐PF4 antibody in the present case. Further functional assay such as the serotonin release assay was necessary to eradicate the suspicion of VITT. Considering myocardial infarction is a common disease in the elderly, MI after vaccination may be just a coincidence. Although the correlation between MI and covid‐19 vaccination could not be made. we should pay attention and do risk evaluation in patients with coronary artery disease before receiving a vaccination. Routine non‐invasive coronary artery examination for high‐risk patients could be a strategy to prevent cardiac complications after vaccination.

We need to be aware of attempts to correlate MI with the vaccine without substantiated data. Any research on the topic should be written carefully and avoid overstating the findings. If more reports of similar serious side effects are published, providers should consider additional screenings prior to COVID‐19 vaccination. Still, the benefits of the vaccines outweigh the risks. This case report would like to emphasize the routine non‐invasive ischemic heart evaluation for the selected high‐risk patient before vaccination.

## FUNDING INFORMATION

The authors have no financial support to disclose.

## CONFLICT OF INTEREST

The authors have no conflict of interest to disclose.

## ETHICAL STATEMENT

All ethical standards were met in writing and submitting this correspondence.

## PATIENT CONSENT STATEMENT

Written informed consent was obtained from the patient for the publication of this case report and accompanying images.

## CLINICAL TRIAL REGISTRATION

N/A.
